# Expression identification of three *OsWRKY* genes in response to abiotic stress and hormone treatments in rice

**DOI:** 10.1080/15592324.2023.2292844

**Published:** 2023-12-18

**Authors:** Jiangdi Li, Yating Chen, Rui Zhang, Bin Wu, Guiqing Xiao

**Affiliations:** College of Bioscience and Biotechnology, Hunan Agricultural University, Changsha, China

**Keywords:** Rice, WRKY transcription factors, stress responses, hormone effects

## Abstract

WRKY transcription factors are critical for plant growth, development, and adaptation to stress. This paper focuses on the expression characteristic to abiotic stress and phytohormones of *OsWRKY24*, *OsWRKY53*, and *OsWRKY70*. Three OsWRKY TFs contained two conserved domains and there were multiple *cis*-elements in response to adversity stress and hormone signaling in their promoters. Real-time PCR analysis revealed their widespread expression in normal tissues during seedling and heading stages. Under various stresses such as darkness, low temperature, salt, and drought, or treatment with hormones like ABA, SA, MeJA, and GA, transcript levels of these genes had changed significantly in wild-type seedlings. The expression level of *OsWRKY24* was upregulated by darkness, cold, SA, and MeJA but downregulated by salt, drought, ABA, and GA treatments. The transcripts of *OsWRKY53* were induced by darkness, low-temperature, salt, drought, ABA, and JA, while inhibited by SA and GA. In addition, *OsWRKY70* expression level was elevated under darkness, low-temperature, SA, and JA but suppressed with salt, drought, ABA, and GA. These findings provide valuable insights into the regulatory mechanisms by which WRKY TFs adapt to stress via plant-hormone signaling.

## Introduction

WRKYs are a class of specific zinc finger-type transcription factors (TFs), which are named after the conserved WRKYGQK domain located at the N-terminal.^1^ WRKY TFs bind to the *cis*-element W-box (T)TGAC(C/T) in the promoter region of target genes and regulate the expression of genes to maintain homeostasis of the internal environment in plants.^2^ Based on the number of domains and the features of zinc fingers, WRKY TFs can be categorized into three subfamilies (group I, -II, and -III). Group I contains two WRKY domains and a zinc finger motif (C-X_4–5_-C-X_22–23_-H-X_1_-H). Group II includes a WRKY domain with the same zinc finger as group I. Group III harbors a domain and a zinc finger structure (C-X_7_-C-X_23_-H-X_1_-C).^1^ The family evolution suggests that all *WRKY* genes might derive from group I.^3^ With the discovery and utilization of more and more functional genes, regulation mechanism of genes has become one of the research focuses.

There were at least 75, 109, and 171 *WRKY* genes had been identified in *Arabidopsis thaliana*, *Oryza sativa*, and *Triticum aestivum*, respectively.^2,4^ WRKY TFs play a pivotal role in the life cycles of plants, including growth, development, senescence, and in the response to various environmental stresses.^5,6^ For instance, AtWRKY71 functioned as a positive regulator in ethylene-mediated leaf senescence by activating *EIN2*, *ORE1*, and *ACS2*.^7^ WRKY1 regulated membrane transporters in *Arabidopsis*, which were involved in stomatal closure to retain water.^8^ OsWRKY28 negatively regulated the transcript of JA biosynthesis genes, thereby impacting root growth.^9^ Overexpression of *OsWRKY76* decreased the resistance of rice blast (*Magnaporthe oryzae*).^10^ Additionally, TaWRKY51 regulated ethylene synthesis and formation of branch roots in wheat by inhibiting the expression of ETH biosynthesis gene *AC*.^11^

Recent research has shown that as a member of WRKY Group I, OsWRKY53, a negative regulator of salt stress, directly trans-regulated expressions of *OsMKK10.2* and *OsHKT1;5*, improving salt tolerance of rice.^12^ In addition, the mutant of *OsWRKY53* enhanced cold tolerance during the booting stage, resulting in a higher yield in rice subjected to cold stress.^13^ We found that the amino acid sequences of OsWRKY24 and OsWRKY70 were 52.43% and 62.87% identities to those of OsWRKY53, respectively. Furthermore, they also belonged to WRKY Group I.^14^ These three TFs had been involved in regulating grain size, and they have been demonstrated to regulate defense responses against fungi, bacteria, and pests.^15–19^ However, it was unclear whether OsWRKY24 and OsWRKY70 had the similar functions to OsWRKY53 in abiotic stress. In this study, we analyzed their conserved motifs and promoter characterization and investigated their expression patterns of tissue-specificity, abiotic stress responses, and hormone effects. These findings provide clues to further unravel the molecular basis and regulatory pathway of OsWRKY TFs under adverse circumstances.

## Results

### Conserved motifs and proteins structure of OsWRKY24, OsWRKY53, and OsWRKY70

**Figure 1.**
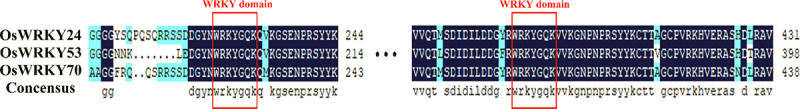
Sequence of the three OsWRKY proteins.

**Figure 2.**
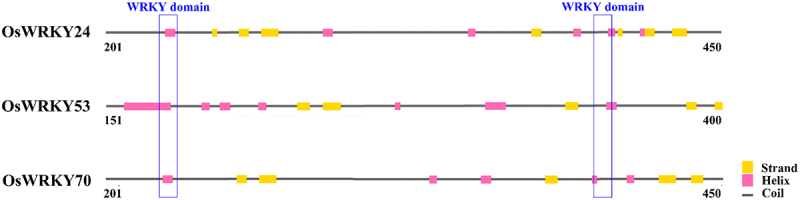
Secondary structure analysis of three OsWRKY proteins.

**Figure 3.**
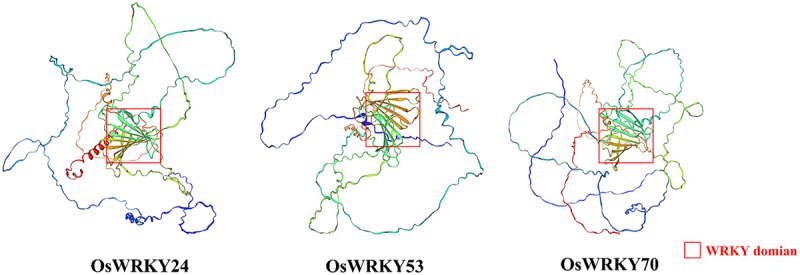
Tertiary structure analysis of OsWRKY proteins.

*Oryza sativa* genes *WRKY24*, -*53*, and -*70* were assigned transcript accession numbers. According to the results of bioinformatics analysis, these proteins were made up of 555, 487, and 572 amino acids, respectively. Their iso-electric points (pI) ranged from 5.52 to 8.05 (Table 1). The number of coils, helix, and strand were distinct in their secondary structures. The WRKY domains which possessed two complete conserved WRKYGQK sequences primarily located in the helix and coils (Figures 1, 2). Furthermore, the tertiary structure analysis revealed that there was a certain similarity in spherical molecule and the conserved domains were positioned at the center of them (Figure 3). The findings suggested that the WRKY TFs may have similar biological functions.

Two conserved WRKYGAK sequences were analyzed at the N-terminal of the three OsWRKY proteins.

Prediction of secondary structure of three OsWRKY transcription factors.

The WRKY domains of the three proteins were similar in structure.

**Table 1.** Basic information of OsWRKY24, OsWRKY53, and OsWRKY70.

Gene-name	MSU_Locus	Amino acids	Location	pI	M.W	GRAVY
*OsWRKY24*	LOC_Os01g61080	555	nucleus	6.61	59305.69	−0.826
*OsWRKY53*	LOC_Os05g27730	487	nucleus	8.05	50980.26	−0.648
*OsWRKY70*	LOC_Os05g39720	572	nucleus	5.52	59652.27	−0.566

### Promoter characterization of OsWRKY24, OsWRKY53, and OsWRKY70

**Figure 4.**
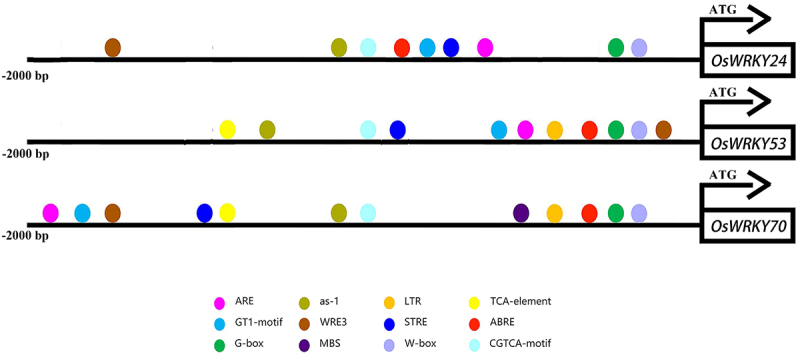
Analysis of promoter elements for *OsWRKY24*, *OsWRKY53*, and *OsWRKY70*.

To elucidate the function underlying the regulation of WRKY TFs, we analyzed the upstream 2000 bp region of *OsWRKY24*, -*53*, and -*70*. The results revealed that they contained a number of core elements which are in response to stress and hormone, such as antioxidant response element (ARE), stress-responsive element (STRE), wound responsive element (WRE3), abscisic acid-responsive element (ABRE), and salicylic acid-responsive element (as-1) (Figure 4). There were 6, 2, and 4 STREs in *OsWRKY24*, -*53*, and -*70*, respectively. Four as-1 elements existed in *OsWRKY24* and seven ABREs in *OsWRKY53* (Table 2). The findings suggest that all these genes may participate in abiotic stress and hormone signaling in rice.

The three *OsWRKY* genes promoter region (2000 bp upstream of the initial ATG) was analyzed. Using online tools (https://bioinformatics.psb.ugent.be/webtools/plantcare/html/), several putative *cis*-regulatory elements related to abiotic stress and hormones were found, including STRE, WRE3, ABRE, and as-1.

**Table 2.** Analysis of promoter elements for OsWRKY24, OsWRKY53, and OsWRKY70.

Upstream elements	Core sequence	Number of elements		
		*OsWRKY24*	*OsWRKY53*	*OsWRKY70*
ARE (antioxidant response element)	AAACCA	3	2	2
G-box (light response elements)	CACGTC	1	5	1
GT1-motif (light response elements)	GGTTAAT	1	1	1
WRE3 (wound-responsive element)	CCACCT	2	1	2
STRE (stress-responsive element)	AGGGG	6	2	4
W-box	TTGACC	4	2	1
ABRE (ABA-responsive element)	ACGTG	1	7	2
as-1 (SA-responsive element)	TGACG	4	1	2
CGTCA-motif (MeJA-responsive element)	CGTCA	4	1	2

### Tissue-specific expression pattern of OsWRKY24, OsWRKY53, and OsWRKY70

**Figure 5.**
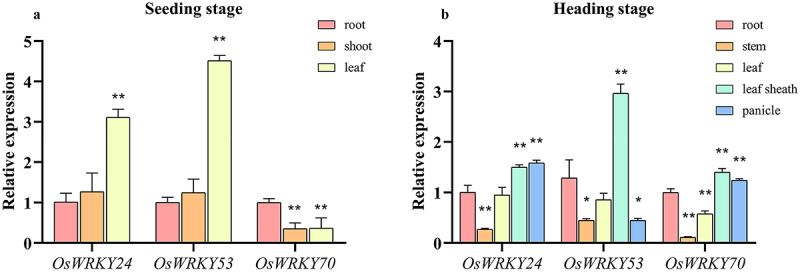
Temporal and spatial expression patterns of *OsWRKY24*, *OsWRKY53*, and *OsWRKY70*.

We examined the expression patterns of *OsWRKY24*, -*53*, and -*70* in WT rice using fluorescence quantitative PCR. The results demonstrated that three *OsWRKY* genes were widely expressed under normal conditions and exhibited distinctive profiles in different tissue parts. At the seeding stage, the high expression level of *OsWRKY24* and *OsWRKY53* were both in leaves, while that of *OsWRKY70* was in the roots which were 3.7 and 4.5 times higher than in shoots and leaves, respectively (Figure 5a). At the heading stage, *OsWRKY24* and *OsWRKY70* showed similar expression patterns in different tissues, they had high transcripts in leaf sheath and panicle, while transcript level of *OsWRKY53* was low in panicle. Three *OsWRKY* genes had low-expression in the stem compared with other organs (Figure 5b). These results imply that three OsWRKY TFs which display tissue-specific expression pattern would play different roles in plant growth and development.

(a) RT-qPCR analysis showing the expression of *OsWRKY* genes in the root, shoot, and leaf of the seeding stage wild-type ‘NIP’. (B) RT-qPCR analysis showing the expression of *OsWRKY* genes in the root, stem, leaf sheath, and panicle of the heading stage wild-type ‘NIP’. Transcript levels in different tissues relative to roots were normalized to the levels of *OsACTIN*. Data are means ± SD (*n*=3 biological replicates). T-test adopts a double-tail test; *: Significant difference (*P*<0.05); **: extremely significant difference (*P*<0.01).

### Expression profiles of OsWRKY24, OsWRKY53, and OsWRKY70 under abiotic stresses

**Figure 6.**
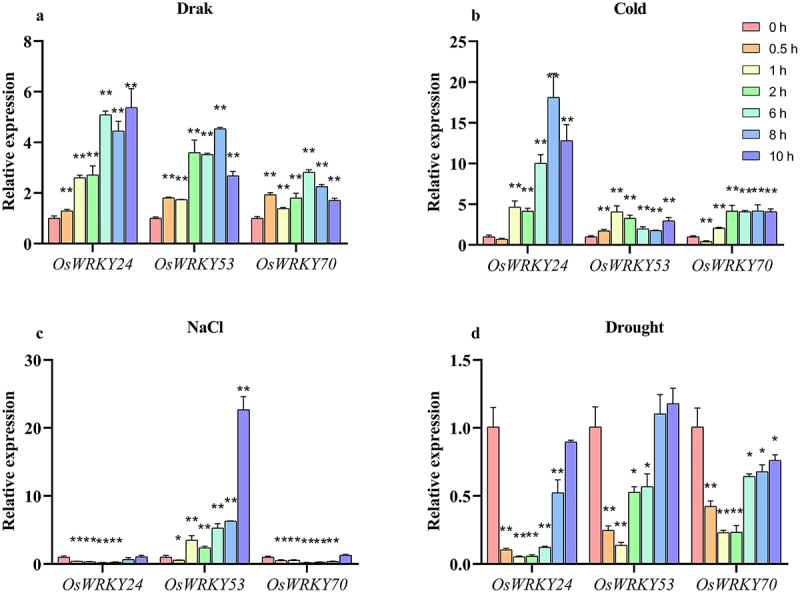
Expression patterns of *OsWRKY24*, *OsWRKY53*, and *OsWRKY70* under dark, cold, salt and simulated drought.

To investigate whether *OsWRKY24*, -*53*, and -*70* involved in stress responses, we conducted transcript levels of these genes under treatment with dark, cold, salt, and drought. The results showed that all three *OsWRKY* genes were significantly induced under dark. High-level transcript of *OsWRKY24* could still be detected at 10 h, which was nearly a 5.5-fold up-regulation, while the expression levels of *OsWRKY53* and *OsWRKY70* reached the peak at 8 h and 6 h then decreased gradually (Figure 6a). Treated with 4°C, the mRNA level of *OsWRKY24* was notably up-regulated nearly 20 times at 8 h. The expression of the three *OsWRKY* genes was clearly induced by cold stress, with a particularly significant up-regulation observed for *OsWRKY24*, which exhibited an approximately 18-fold increase at 8 h (Figure 6b). Three *OsWRKY* genes were also affected differentially by high salinity condition. As shown in Figure 6c, the salt induction of *OsWRKY53* gradually increased during the early stages of stress, peaking at 10 h (22-fold). By contrast, *OsWRKY24* and *OsWRKY70* were constantly repressed from 0.5 h to 6 h and then rose to normal level by salt stress. The expression profiles of the three *OsWRKY* genes were significantly inhibited by drought stress, their mRNA levels reached the lowest level at 1 h and then increased gradually (Figure 6d). Taken together, these data indicate that three OsWRKY TFs might play an important role in response to different abiotic stresses.

(a) RT-qPCR analysis showing the dark-induced expression of the three *OsWRKY* genes. (b) RT-qPCR analysis showing the cold-induced expression of the three *OsWRKY* genes. (c) RT-qPCR analysis showing the salt-induced expression of the three *OsWRKY* genes. (d) RT-qPCR analysis showing the simulated drought-induced expression of the three *OsWRKY* genes. Total RNA was extracted from the leaves 2-week-old ‘NIP’ plants at 0, 0.5, 1, 2, 6, 8, and 10 h of abiotic stresses. Transcript levels relative to 0 h (prior to treatment) for each time point were normalized to the level of *OsACTIN*. Data are means ± SD (*n*=3 biological replicates). T-test adopts a double-tail test; *: Significant difference (*P*<0.05); **: extremely significant difference (*P*<0.01).

### Expression profiles of OsWRKY24, OsWRKY53, and OsWRKY70 under hormone treatments

**Figure 7.**
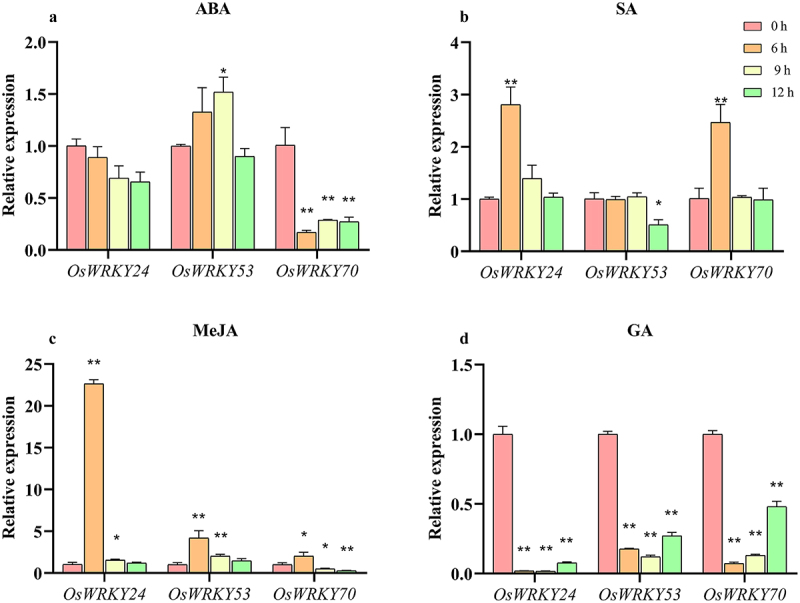
Expression patterns of *OsWRKY24*, *OsWRKY53*, and *OsWRKY70* responses to ABA, SA, MeJA and GA.

To study whether three *OsWRKY* genes in response to hormone, we further performed ABA, SA, MeJA, and GA on leaves of two-week-old seedings. The results showed that *OsWRKY24* and *OsWRKY70* were both suppressed by ABA, the latter exhibited more sensitivity to ABA, with approximately sixfold down-regulation at 6 h (Figure 7a). On the contrary, the expression level of *OsWRKY53* was induced by ABA, which reached the peak at 9 h and returned to the normal level at 12 h (Figure 7a). The transcripts of *OsWRKY24* and *OsWRKY70* were induced by treatment of SA, with about a 3-fold and 2.5-fold upregulation at 6 h, respectively. In contrast, *OsWRKY53* responded to SA slowly, which was inhibited at 12 h (Figure 7b). All three *OsWRKY* genes were induced by MeJA. Among them, *OsWRKY24* was particularly sensitive to MeJA, with an over 20-fold up-regulation at 6 h (Figure 7c). In addition, under GA treatment, they were all repressed significantly within 6 h followed by maintenance of low transcript levels (Figure 7d). These results strongly suggest that these OsWRKY TFs may be involved in regulatory effects of phytohormone signaling.

(a) RT-qPCR analysis showing the ABA-induced expression of the three *OsWRKY* genes. (b) RT-qPCR analysis showing the SA-induced expression of the three *OsWRKY* genes. (c) RT-qPCR analysis showing the MeJA-induced expression of the three *OsWRKY* genes. (d) RT-qPCR analysis showing the simulated GA-induced expression of the three *OsWRKY* genes. Total RNA was extracted from the leaves 2-week-old ‘NIP’ plants at 0, 6, 9, and 12 h of hormone treatments. Transcript levels relative to 0 h (prior to treatment) for each time point were normalized to the level of *OsACTIN*. Data are means ± SD (*n*=3 biological replicates). T-test adopts a double-tail test; *: Significant difference (*P*<0.05); **: extremely significant difference (*P*<0.01).

## Discussion

It is well known that the structure of a protein determines its function. The diverse biological functions of protein molecules are based on their extremely complex chemical composition and spatial structure. Primary and three-dimensional structures of proteins are closely related to their specific functions. The WRKY domain is capable of specifically binding to the promoter W-box motif (T)(T)TGAC(C/T), where TGAC represents the core conserved sequence of W-box and is directly associated with the specificity of WRKY transcription factors and their downstream targets.^20^ Two conserved WRKY domains exist in OsWRKY24, −53, and −70, in which the coding amino acid was between the 200th and the 450th (Figures 1–3), indicating that their membership in WRKY group I. In *Arabidopsis*, WRKY18, WRKY40, and WRKY60 are very related in structure, which exhibit partially functional redundancy as negative regulators in plant resistance against the biotrophic bacterial pathogen *P. syringae* and the fungal pathogen *E. cichoracearum*.^21^ *AtWRKY38* and *AtWRKY62*, which encode two similar-type group III WRKY transcription factors, exert a regulatory role in the defense response against *P. syringae*.^22^ In addition, GmWRKY58 and GmWRKY76, which belong to WRKY Group III, play a critical role in plant growth and flowering.^24^ In recent research, it has been reported that OsWRKY24, −53, and −70 were involved in regulation of grain size.^18,25^ We speculate that the specific expression of OsWRKY24, −53, and −70 in panicles at the heading stage may be related to the regulation of grain size (Figure 5b). Moreover, OsWRKY24 and OsWRKY53 play an important role in the regulation of blast resistance.^17,23^ OsWRKY53 and OsWRKY70 as a specific herbivore-induced, activating the rice defense response against herbivores.^16,26^ These findings imply that WRKY proteins may display functional similarity in closely structural homologs.

Abiotic stresses, such as temperature inversion, salinity, and drought adversely affect the physiological and biochemical processes of plants.^27^ Plants always modulate their gene expression in response to changes in the surrounding environment, which might thereby fine-tune the relevant physiological functions of proteins. Previous studies have demonstrated that WRKY TFs are involved in the regulation of plant growth and stress responses.^2^ OsWRKY53 has been reported to play a critical role in leaf senescence,^28^ In this paper, we found that promoter regions of *OsWRKY24*, -*53*, -*70* contained 2, 6, and 2 light-responsive elements, respectively. Expressions of these genes were activated by darkness, and it seems that OsWRKY24 and OsWRKY70 could be possibly senescence-induced transcription factors. OsWRKY53 has been demonstrated to negatively regulate cold tolerance at the booting stage. OsWRKY24 is a potential candidate gene to influence the cold sensitivity of ‘Towada’ and ‘ZL31’.^13,29,30^ Moreover, the analysis also showed that three *OsWRKY* genes were induced by low temperature (Figure 6b). Our experiments preliminarily found that the *oswrky70* was sensitive to cold stress (no published data). The functions of OsWRKY24 and OsWRKY70 in chilling tolerance need to be further clarified. We found that *OsWRKY53* was induced by salt strongly, while *OsWRKY24* and *OsWRKY70* were repressed (Figure 6c). OsWRKY53-OsMKK10.2 and OsWRKY53-OsHKT1;5 modules coordinate to regulate iron stress, which has been demonstrated.^12^ *OsWRKY53* was upregulated by the salt environment, which was consistent with the results reported above, suggesting OsWRKY24 and OsWRKY70 may play a regulatory role in the salt stress response. Regarding PEG treatment, *OsWRKY24*, -*53*, and -*70* were also downregulated (Figure 6d), indicating their involvement in the response to drought stress. However, further investigation is needed to elucidate the specific regulatory mechanisms among these genes in the future.

Phytohormone is necessary for understanding the significance of WRKY TFs in plant development and stress. In *Arabidopsis*, WRKY62 could interact downstream of NPR1 and negatively regulate expression of JA-responsive genes, suggesting its involvement in the SA-mediated suppression of JA signaling.^31^ Rice OsWRKY50 has regulated the ABA-dependent seed germination, seedling growth, and salt tolerance.^32^ We found that the promoter of the three *OsWRKY* genes contained lots of *cis*-elements of hormone response (Figure 4). Three *OsWRKY* genes were suppressed by GA, *OsWRKY24* and *OsWRKY70* was repressed by ABA, while *OsWRKY53* was induced by ABA (Figure 7a-d). These three WRKY TFs have been speculated that may function in a partially redundant manner to antagonize the ABA and GA signaling pathways.^14^ Moreover, OsWRKY53 has been proven to directly bind the promoters of *OsABA8ox1* and *OsABA8ox2* to repress their expression, resulting in elevated endogenous ABA contents that promoted leaf senescence.^28^ Meanwhile, OsWRKY53 regulates the cold tolerance by suppressing the expression of gibberellin (GA) biosynthesis genes and reducing GA content in anther.^13^ Therefore, it is worth considering whether the OsWRKY24 and OsWRKY70 are also involved in the response to abiotic stress through phytohormone signaling pathways. The conventional model of JA and SA signaling crosstalk involves antagonism between these two hormones.^33^ *OsWRKY24* and *OsWRKY70* were induced by MeJA and SA, while transcript level of *OsWRKY53* was suppressed by SA and increased by MeJA (Figure 7b,c), altered expression of *OsWRKY70* has been found to affect the content of SA, JA, and GA.^16^ OsWRKY24 as a blast-disease responsive transcription factor, positively regulates rice disease resistance; OsWRKY53 has also been reported to be involved in the regulation of bacterial and fungal resistance,^17,19,23,34,35^ further investigation is required to confirm the role, if any, of OsWRKY24, −53, and −70 in the crosstalk between SA, JA, and biotic stress response in rice knockout mutants.

Plant growth, development, and responses to stress are regulated by a complex and intricate network, where different genes and hormones may exhibit synergistic or antagonistic effects. Although the three *OsWRKY* genes exhibit different expression patterns in response to stress and hormones, overall, they represent a self-defense mechanism employed by plants to cope with adverse environments. Continued exploration of the expression patterns of various genes under different stress conditions will provide clues for understanding their functions and underlying mechanisms.

## Materials and methods

### Plant materials, growth condition, and treatments

Rice seeds ‘NIP’ (*Oryza sativa L. japonica*) were imbibed in water in the dark at 37°C for 2 days. Germinated seeds were planted in substrate soil and grown in natural conditions and irrigated with 1/2 Yoshida culture under controlled light conditions (28°C, 16 h of light and 8 h of darkness).

To collect different parts of rice plants at different stages, including 2-week-old rice (roots, shoots, and leaves) and the heading stage (roots, stems, leaves, leaf sheaths, and panicles), 2-week-old rice seedlings were used for stress and hormone treatment. After 2 weeks, the seedlings were used for the following seven treatments. For darkness, seedlings were placed in a dark box. For cold treatments, seedlings were exposed to 4°C (16 h of light and 8 h of darkness). For salt stress and drought stress, seedlings were irrigated with 1/2 Yoshida culture containing 0.6% NaCl and 10% polyethylene glycol (PEG 6000), respectively. For ABA, JA, SA, and GA treatment, seedlings were sprayed 0.4 mmol/L ABA, 1 mmol/L SA, 100 μmol/L MeJA, and 100 μmol/L GA_3_, respectively. For stress treatment, the leaves of the samples were collected at 0, 0.5, 1, 2, 6, 8, and 10 h separately. For hormones treatment, samples were collected at 0, 6, 9, and 12 h separately. Each treatment was repeated three times.

### Analysis of basic information and cis-elements of OsWRKY24, OsWRKY53, and OsWRKY70

The whole genome data of *Oryza sativa L. japonica* come from RGAP (http://rice.uga.edu/) database. Multiple sequence alignment of OsWRKY24, OsWRKY53, and OsWRKY70 identified was carried out using DNAMAN 8. PSIPRED (http://bioinf.cs.ucl.ac.uk/psipred/) and SWISS-MODEL (https://swissmodel.expasy.org/interactive) were used to analyze the secondary structure and the tertiary structure of three WRKY transcription factors. Upstream 2000-bp promoter sequences of these *OsWRKY* genes were obtained from NCBI (https://www.ncbi.nlm.nih.gov/) that were submitted to Plant CARE (https://bioinformatics.psb.ugent.be/webtools/plantcare/html/) for predicted *cis*-elements.

### Total RNA extraction, reverse transcription, and qPCR analysis

For gene expression analysis, total RNA from the leaves of treated samples was isolated using Ultrapure RNA 200 preps Kit (Cwbio, China). cDNAs were synthesized with the HiScript III 1st Strand cDNA Synthesis Kit (+gDNA wiper) (Vazyme, China) according to the manufacturer’s protocol. Quantitative real-time PCR (RT-qPCR) was performed using ChamQ Universal SYBR qPCR Master Mix (Vazyme, China) in the CFX Connect Real-Time PCR Detection System (Bio-Rad, United States) as 95°C for 5 min, 95°C for 10 s, 60°C for 30 s, 40 cycles, and three replicates per sample. Gene-specific primers were designed using NCBI. The transcript levels of rice actin gene were used to normalize expression levels for genes (Table 3). The transcript levels of the examined genes were quantified by a relative quantitation method (2^−▲▲T^). Each RT-qPCR assay was biologically repeated at least twice with similar results, with each repetition having three technical replicates. Microsoft® Excel was used for data analysis. The differences were analyzed for statistical significance using two-tailed Student’s t-test.

**Table 3.** The list of qPCR primers used in the study.

qPCR primers	5’—>3’
*OsACTIN*-F	GGCATTGCTGACAGGATGAG
*OsACTIN*-R	GCTTAGCATTCTTGGGTCCG
*OsWRKY24*-q-F	TGCTCCTGACCTCCAGTATC
*OsWRKY24*-q-R	GCTGCTGACTTCTGTATGCC
*OsWRKY53*-q-F	GAGCGACATCGACATCCT
*OsWRKY53*-q-R	TTGTGCTTGCCCTCGTAG
*OsWRKY70*-q-F	TACTCTTACACGAGCCAGCA
*OsWRKY70*-q-R	ATTGACGGCCCGATTAGATG

### Primers

The qPCR primers as shown in (Table 3) were designed using Snapgene software and synthesized by Beijing Tsingke Biotech Co., Ltd.

## Data Availability

Data is contained within the article or supplementary material.
